# Evolution of B Chromosomes: From Dispensable Parasitic Chromosomes to Essential Genomic Players

**DOI:** 10.3389/fgene.2021.727570

**Published:** 2021-12-09

**Authors:** Martina Johnson Pokorná, Radka Reifová

**Affiliations:** ^1^ Department of Zoology, Charles University, Prague, Czech Republic; ^2^ Department of Ecology, Charles University, Prague, Czech Republic; ^3^ Institute of Animal Physiology and Genetics, Czech Academy of Sciences, Liběchov, Czech Republic

**Keywords:** evolution, cytogenetics, supernumerary chromosomes, meiotic drive, cellular domestication

## Abstract

B chromosomes represent additional chromosomes found in many eukaryotic organisms. Their origin is not completely understood but recent genomic studies suggest that they mostly arise through rearrangements and duplications from standard chromosomes. They can occur in single or multiple copies in a cell and are usually present only in a subset of individuals in the population. Because B chromosomes frequently show unstable inheritance, their maintenance in a population is often associated with meiotic drive or other mechanisms that increase the probability of their transmission to the next generation. For all these reasons, B chromosomes have been commonly considered to be nonessential, selfish, parasitic elements. Although it was originally believed that B chromosomes had little or no effect on an organism’s biology and fitness, a growing number of studies have shown that B chromosomes can play a significant role in processes such as sex determination, pathogenicity and resistance to pathogens. In some cases, B chromosomes became an essential part of the genome, turning into new sex chromosomes or germline-restricted chromosomes with important roles in the organism’s fertility. Here, we review such cases of “cellular domestication” of B chromosomes and show that B chromosomes can be important genomic players with significant evolutionary impact.

## Introduction

B chromosomes are supernumerary dispensable chromosomes that occur only in some individuals or populations within a species, or only in a subset of cells or tissues within an individual (Beukeboom, 1994; Camacho, 2005; Houben et at., 2014; Ruban et al., 2020). Their presence in a species can thus be viewed as a type of genetic polymorphism. Unlike standard chromosomes, they often show irregular non-Mendelian inheritance (Jones, 1995). B chromosomes were observed for the first time by Wilson (1907) in true bugs (Hemiptera) from the genus *Metapodius*. Soon after, similar observations were published for cucumber beetles (Coleoptera) from the genus *Diabrotica* (Stevens, 1908). In plants, such structures were first observed in rye (*Secale cereale*) and named “k-chromosomes” by Gotoh (1924), and later described in maize (*Zea mays*) by Kuwada (1925) and Longley (1927) who labelled them as “supernumerary chromosomes.” The term “B chromosomes” was introduced by Randolph (1928) and has been used by the scientific community ever since. In synchrony, all the other chromosomes in the genome are referred to as “A chromosomes”.

Based on the number of species studied so far it has been estimated that approximately 15% of eukaryotic species have B chromosomes (Beukeboom, 1994; D’Ambrosio et al., 2017) with new findings of B chromosomes being regularly described (reviewed in Jones, 2017). A database collecting information about B chromosomes has been available since 2017 (D’Ambrosio et al., 2017). Out of the 2,828 eukaryotic species with B chromosomes reported there, 73.56% are plants, 25.95% animals and 0.49% fungi (D’Ambrosio et al., 2017). However, it is important to note that some groups of organisms have been cytogenetically studied less extensively than others and thus the representation of B chromosomes among specific taxonomic groups is currently difficult to compare (D’Ambrosio et al., 2017).

B chromosomes can occur in single or multiple copies per cell but usually their copy number is rather low. There is normally either one or a few copies of B chromosomes functioning usually as univalents (Camacho, 2005). However, in some species, extreme numbers of B chromosomes can be observed in a single cell, such as in some plant species of the genus *Pachyphylum* (Crassulaceae), which have up to 50 B chromosome copies (Uhl and Moran, 1973). Other organisms with high numbers of B chromosomes are maize (*Z. mays*) with up to 34 copies (Jones and Rees, 1982), the wood mouse (*Apodemus peninsulae*) with up to 24 copies (Volobuev and Timina, 1980) and the *Xylota nemorum* fly with up to 24 B chromosomes copies (Boyes and van Brink, 1967). In some cases, extensive variability in the number of B chromosomes can be observed among individuals or populations within a species (Camacho et al., 2000). It has been reported that these high numbers of B chromosomes could have negative effects on their hosts, particularly on their fertility and viability (Houben, 2017), especially if the B chromosomes occur in odd numbers (Camacho et al., 2004).

B chromosomes show high variability in their size across species. They can be similar in size to A chromosomes (Jones, 2018) but also, in some species, B chromosomes are considerably smaller than the smallest A chromosomes e.g., in the harvest mouse *Reithrodonomys megalotis* (Peppers et al., 1997) or the fly *Megaselia scalaris* (Wolf et al., 1991). On the other hand, B chromosomes bigger than the biggest A chromosomes have been reported in cyprinid fish *Alburnus alburnus* (Ziegler et al., 2003), the giant white-tailed rats *Uromys caudimaculatus* (Baverstock et al., 1982) and the characid fish *Aslyanax scabripinnis* (Mestriner et al., 2000). Variation in size can also be observed within a species e.g. in the grasshopper *Eyprepocnemis plorans* (López-León et al., 1993).

B chromosomes were for a long time considered to have no important function for the carrier individual and spread mostly as genome parasites (e.g., Ӧstergren, 1945; Van Valen, 1977). Currently, however, the view of B chromosomes is changing with many active B chromosome genes with important functions for their hosts being discovered (reviewed in Houben et al., 2014; Ruban et al., 2017, Dala Benetta et al., 2020). Based on these findings, it has been proposed that the effects of B chromosomes on their host may shift back and forth from parasitic or neutral to beneficial (Camacho et al., 2000). In some species, it has been even hypothesised that B chromosomes may became an essential, stable part of the genome turning, for example, into new sex chromosomes or chromosomes restricted to germline that became essential for viability and fertility of their carriers (Carvalho, 2002; Nokkala et al., 2003; Dalíková et al., 2017; Torgasheva et al., 2019; Imarazene et al., 2021; Lewis et al., 2021). In this review we describe mechanisms of B chromosome origin, strategies of their inheritance and give examples of the “cellular domestication” of B chromosomes, where these chromosomes provide some important functions for their hosts. We reviewed possible pathways of B chromosome evolutionary dynamics with outcomes ranging from the classical view of B chromosomes as nonessential genetic elements spreading in the population as genomic parasites to important genomic players providing benefits to their hosts.

### Origin of B Chromosomes

The question of where B chromosomes come from has been puzzling researchers since their discovery. Currently, the most likely explanation is that B chromosomes originate from A chromosomes as by-products of chromosomal rearrangements or unbalanced segregation, when a chromosome fragment or an extra copy of an A chromosome may develop into a proto-B chromosome (Peters, 1981; Jones and Rees, 1982; Talavera et al., 1990; Camacho et al., 2000; Mestriner et al., 2000; Dhar et al., 2002; Bauerly et al., 2014). Once a proto-B chromosome exists it may acquire additional genetic material through duplications from other A chromosomes (Martis et al., 2012; Marques et al., 2018; Ahmad et al., 2020; Blavet et al., 2021). Throughout the evolution of B chromosomes, various mobile elements and unique coding and noncoding sequences can be incorporated, sometimes amplifying and sometimes degenerating due to the very small selection pressure on the B chromosome (reviewed in Houben et al., 2014; Marques et al., 2018). Next-generation sequencing of B chromosomes in various species confirmed that these chromosomes are largely composed of A chromosome paralogous sequences, although in some plant species organellar DNA has also been shown to contribute to the B chromosomes (Valente et al., 2014; Ruban et al., 2017; Marques et al., 2018; Ruiz Ruano et al., 2018). For example, in rye (*S. cereale*), one of the best-studied plant models for B chromosome research, B chromosomes seem to harbor A chromosome derived sequences, mostly coming from 3R to 7R autosomes, with a significant contribution of organellar DNA (Martis et al., 2012). Similarly, in the goat grass *Aegilops speltoides,* B chromosomes share sequences not only with A chromosomes but also with the DNA of plastids and mitochondria, suggesting that organelle-to-nucleus DNA transfer affects B chromosome evolution (Ruban et al., 2014; Ruban et al., 2020). The level of homology between B chromosomal and A chromosomal paralogous sequences can be used to estimate the age of B chromosomes. In maize (*Z. mays*), such comparison revealed the very ancient origin of the B chromosomes (Blavet et al., 2021).

In several species, B chromosomes appear to have originated from sex chromosomes. This seems to have happened for example in the flies from the genus *Glossina*, the New Zealand endemic frog *Leiopelma hochstetteri* (reviewed in Camacho, 2005) and the grasshopper *E. plorans,* where the B chromosome is derived from a paracentromeric region of the X chromosome (López León et al., 1994). The involvement of sex chromosomes in the origin of B chromosomes has been also demonstrated in the rodent group Oryzomyini (Ventura et al., 2015).

Although it seems to be rare, B chromosomes may also originate through interspecific hybridization. This has been described for jewel wasps *Nasonia vitripennis*. This species-specific B chromosome known as Paternal Sex Ratio (PSR) contains transposon-like sequences that appear to be absent from the *N. vitripennis* genome, but match sequences present in another two species of wasp from the genus *Trichomalopis* (McAllister, 1995; McAllister and Werren, 1997). This observation suggests that this B chromosome has been derived from a chromosome of another species that moved into the *N. vitripennis* genome by hybridization (McAllister, 1995; McVean, 1995; McAllister and Werren, 1997; Perfectti and Werren, 2001).

B chromosomes may also have their origin in incompletely expelled A chromosome from the sperm in pseudogamous parthenogens as documented in flatworms *Polycelis nigra*. In this species, sexual individuals are always diploid while pseudogamous parthenogens are usually triploid. In parthenogenetic individuals a sperm is required only to initiate the egg development and the paternal chromosomes never enter the oocyte nucleus. In purely parthenogenetic populations of this species, B chromosomes of three distinct subtypes were found in almost all individuals. These B chromosomes come from paternal A chromosomes which escaped the exclusion of the sperm genome and have been incorporated into the nucleus (Beukeboom et al., 1996; Sharbel at al., 1997).

### B Chromosomes as Genomic Parasites

Traditionally, B chromosomes have been viewed as genomic parasites that do not provide any advantage to their host and sometimes can even be detrimental if they are present in high copy numbers (Camacho et al., 2004). Because their meiotic as well mitotic inheritance may be unstable especially if they occur in an odd copy number, B chromosomes evolved diverse ways to promote their own transmission, preventing their loss from the population. These include meiotic drive, mitotic drive associated with gonotaxis and preferential fertilization of the ovum by B chromosome carrying spermatozoa.

**FIGURE 1:**
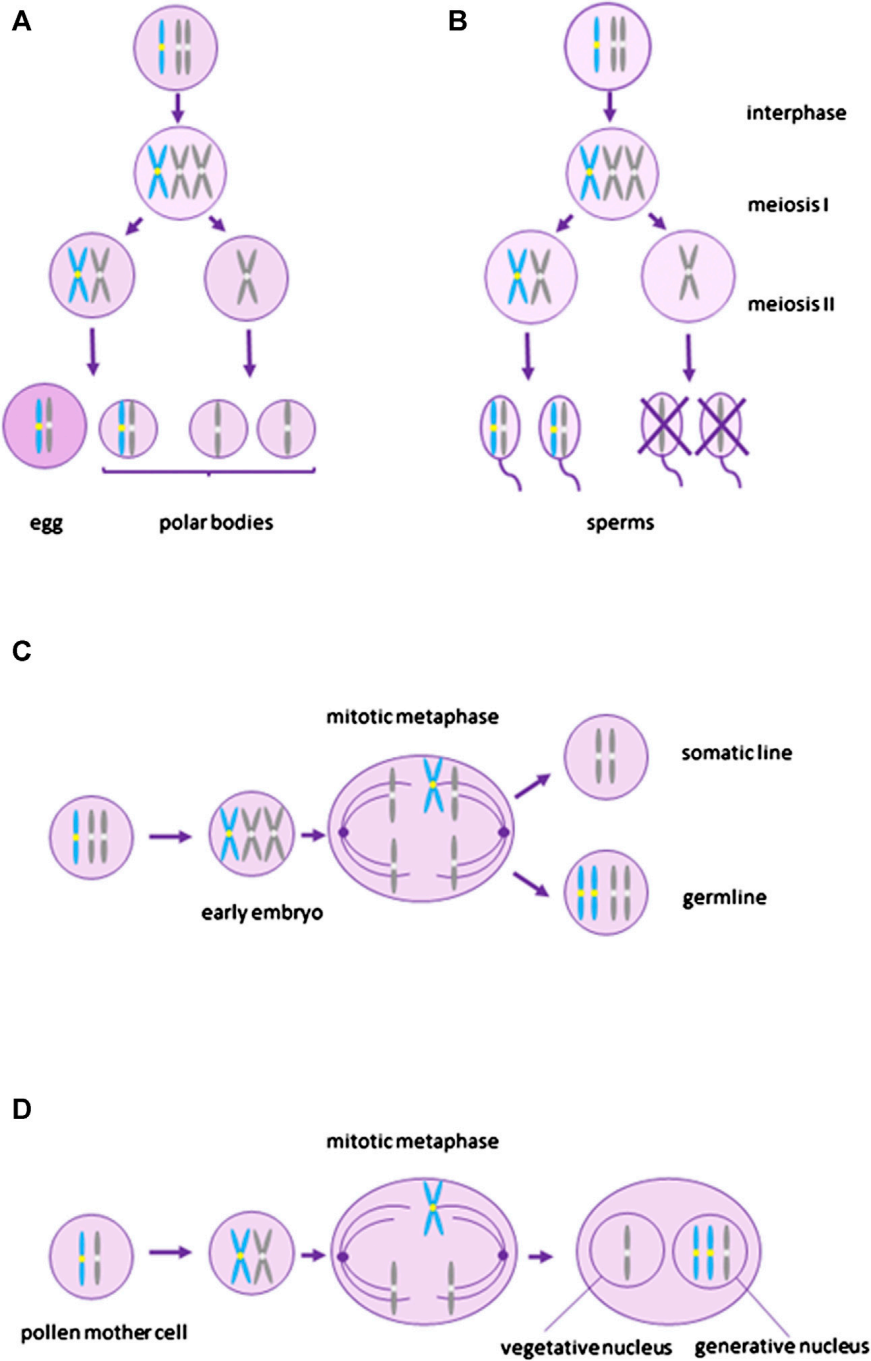
Depiction of transmission mechanisms of B chromosomes. **(A)** and **(B)** represent meiotic drive. **(A)**–female meiotic drive where the B chromosome segregates preferentially into the egg, **(B)**–male meiotic drive where sperms without B do not survive. **(C)** and **(D)** represent mitotic drive associated with gonotaxis where B chromosomes preferentially segregate into the germline **(C)**–premeiotic mitotic drive during early embryo development when the germline is being determined, **(D)**–postmeiotic mitotic drive in plants during gametophytic phase. Blue represents B chromosomes and grey represents A chromosomes.

Meiotic drive (Figure 1A,B) promoting the transmission of B chromosomes can occur during female as well as male meiosis. Female meiotic drive (Figure 1A) is, however, more common (Jones, 2018). It stems from the asymmetry of female meiosis, where one ovum and three polar bodies (which do not participate in inheritance) are produced from a single diploid oogonia. Many B chromosomes have been shown to have a mechanism helping them to end up in the ovum rather than in a polar body (reviewed in Jones, 2018). Female meiotic drive is often associated with specific centromeric sequences, as centromeres bind to the meiotic spindle during chromosome segregation (Padro-Manuel De Villena and Sapienza, 2001). However, sometimes the total number of centromeres in each side of the meiotic spindle, rather than specific centromeric sequences, determine which chromosomes will end up in the egg and which in polar bodies (Padro-Manuel De Villena and Sapienza, 2001). If a higher number of centromeres preferentially segregate to the egg, the presence of an additional B chromosome bringing one extra centromere, may cause B chromosomes to preferentially segregate to the gamete. Generally, such a type of meiotic drive based on the total number of centromeres may also lead to a chromosome fission and the origin of acrocentric chromosomes from metacentric chromosomes (Padro-Manuel De Villena and Sapienza, 2001. Interestingly, it has been observed in mammals and insects that B chromosomes occur more often in species with acrocentric rather than metacentric chromosomes (Bidau and Martí, 2004; Palestis et al., 2004; Palestis et al., 2010), suggesting that female meiotic drive based on total number of centromeres may help to spread B chromosomes in a population. However, we cannot rule out the possibility that acrocentric chromosomes simply generate B chromosomes more frequently than metacentric chromosomes.

B chromosomes can also increase their transmission by male meiotic drive (Figure 1B), where haploid cells without B chromosomes do not survive, although it seems to be much rarer than female meiotic drive (Jones and Rees, 1982). A specific case of male meiotic drive was described in the mealybug *Pseudococcus affinis.* In this species, paternally inherited chromosomes become heterochromatic during early embryonic development. During male meiosis, paternal and maternal chromosomes segregate to different poles and only meiotic products with euchromatic maternal chromosomes form functional sperm. The B chromosome, although paternally inherited, segregates with the maternal chromosomes and ends up in the functional sperm (Nur, 1962).

Mitotic drive leading to the preferential segregation of B chromosomes to the germline (gonotaxis) (Figure 1C,D) may also increase the chance of B chromosome transmission to the next generation. At the same time, it can lead to the multiplication of B chromosome copies in the cell. Mitotic drive occurs through the nondisjunction of B chromosomes during mitosis (Jones, 1991). It can happen before meiosis during the germline cell division and in plants also after meiosis during the gametophytic phase. Premeiotic mitotic drive (Figure 1C) was described for the first time in the grasshoppers *Calliptamus palaestinensis* and *Cammula pelucida* (Nur, 1963; Nur, 1969) and leads to an amplification of B chromosomes in spermatocytes. Similar phenomenon was later observed in other animals and plants (Rutishauser and Rӧthlisberger, 1966; Kayano, 1971; Viseras et al., 1990; Pardo et al., 1995; Jones, 2018). The postmeiotic mitotic drive (Figure 1D) is known from plants which have, compared to animals, a haploid gametophyte phase (reviewed by Houben, 2017). This type of drive was first observed by Hasegawa (1934) in rye, where the B chromosome moved with its two nondisjunct chromatids towards the generative nucleus in the first pollen grain mitosis. The nondisjunction in rye is controlled by the region on the long arm of the B chromosome where various tandem repeats have been identified (reviewed in Houben et al., 2014; Marques et al., 2018).

In some plants, the overtransmission of B chromosomes can be caused by preferential fertilization of the ovum by the B-carrying spermatozoid (Jones, 2018). This has been observed for example in maize, where mitotic drive at the second pollen mitosis caused by nondisjunction of B chromosome chromatids results in two unequal sperms. The egg is then preferentially fertilized by the sperm bearing the B chromosome during double fertilization (Blavet et al., 2021).

Although different mechanisms of unequal transmission already play very powerful roles in B chromosome inheritance, some B chromosomes found a way to enhance the drive effect. For example, some cichlid fish from Lake Victoria and Malawi carry a female-specific B chromosome (Yoshida et al., 2011; Clark and Kocher, 2019), which exhibits meiotic drive ending up in more than half of oocytes. Interestingly, offspring of B-transmitting females show a strong female biased sex ratio and genotyping of these offspring revealed that the B chromosome carries a female sex determining gene that is epistatically dominant to an XY system. Therefore, the outcome is that an XY fish with a B chromosome becomes a female and an XY fish without a B chromosome becomes a male. It has been suggested that the new sex determining function of the B chromosome evolved to enhance the female meiotic drive of the B chromosome without providing any beneficial effect to the host (Clark and Kocher, 2019).

Another example of the B chromosome, which can manipulate the sex of its carrier to enhance its own transmission, is PSR known from the jewel wasp *N. vitripennis* and other haplodiploid arthropods (Werren et al., 1987; Werren, 1991; Werren and Beukeboom, 1993; Werren and Stouthamer, 2003). PSR is transmitted strictly paternally and causes the complete elimination of the paternal chromosomes (except for the PSR itself) after fertilization (Reed and Werren, 1995; Dalla Benetta et al., 2020). As a result, all diploid fertilized eggs which would normally develop into females are turned into males causing an extremely male biased sex ratio (Werren and Beukeboom, 1993; Werren and Stouthamer, 2003).

B chromosomes might also be involved in the genetic control of apomixis (e.g., asexual reproduction via seeds). This has been described in *Boechera stricta* and *B. holboellii* (Brassicaceae). In these species, there is an additional *Het* chromosome which in some cases went through fission resulting in a largely heterochromatic *Het’* chromosome in all apomictic individuals and a smaller *Del* chromosome in aneuploid apomictic individuals. Because these chromosomes are present exclusively in apomictic plants, it has been suggested that they could play a role in the genetic control of apomixis (Sharbel et al., 2004; Kantama et al., 2007; Mandáková et al., 2015). As asexual reproduction can ensure maintaining the stable combination of chromosomes, B chromosomes involved in the transition to asexuality could theoretically gain advantage in their own transmission. However, Mandáková et al. (2021) argued that these chromosomes may be more a consequence of apomixis rather than its cause.

New genomic approaches enabling the sequencing of B chromosomes allow the identification of specific genes and regions on the B chromosome causing the drive (Dalla Benetta et al., 2020). A nice example of a gene involved in its own drive is the *haploidizer* located on PSR in *N. vitripennis* which is responsible for the elimination of paternal chromosomes except for PSR (Dalla Benetta et al., 2020). Banaei-Moghaddam et al. (2012) and Banaei-Moghaddam et al. (2013) identified the B-specific centromeric sequence responsible for the extended cohesion of the B chromatids during the first pollen mitosis and their preferential segregation to the generative nucleus in rye. Using whole B chromosome assembly, Blavet et al. (2021) determined regions on the maize B chromosome including B chromosome-specific repeat concentrated around the centromere and *trans*-acting factors on the long arm involved in B chromosome nondisjunction at the second pollen mitosis. Interestingly, centromeric region also played a role in a preferential fertilization of the egg by sperm carrying B-specific centromere (Blavet et al., 2021). In addition, in several organisms, genes involved in cell cycle, cell division, chromosome segregation or cell differentiation have been identified on B chromosomes (Graphodatsky et al., 2005; Makunin et al., 2016; Navarro Domínguez et al., 2017; Makunin et al., 2018; Marques et al., 2018; Kichigin et al., 2019; Martins and Jehangir, 2021). These might represent candidate genes for B chromosome drive. The origin of such sequences promoting the B chromosome transmission is assumed to play an important role during early B chromosome evolution (Houben et al., 2014). Without such sequences the B chromosome is likely destined to be lost from the population if it does not provide any advantage to the host (Camacho et al., 2000; Ahmad et al., 2020).

### Cellular Domestication of B Chromosomes

As described in the previous section, for a long time B chromosomes were mostly viewed as nonessential selfish elements which had either no effect on the host or a negative one, and spread through the population solely as genome parasites. However, there is accumulating evidence that B chromosomes in many organisms carry important functions for their hosts, which can help them to spread in the population without selfish mechanisms such as drive (Table 1).

**TABLE 1:** List of examples where B chromosomes have a beneficial or necessary function for their hosts.

Species	Group	Function	Reference
*Nectria haematococca*	fungus (Ascomicota)	resistance to antibiotics	Miao et al. (1991)
			Enkerli et al. (1997)
		increased pathogenicity on pea roots	Rodriguez-Carres et al. (2008)
		utilization of unique carbon/nitrogen sources	Coleman et al. (2009)
*Magnaporthe oryzae*	fungus (Ascomicota)	increased pathogenicity	Ma et al. (2010)
			Balesdent et al. (2013)
			van Dam et al. (2017)
*Fusarium oxysporum*	fungus (Ascomicota)	increased pathogenicity	Armitage et al. (2018)
			Balesdent et al. (2013)
			Ma et al. (2010)
			Thatcher et al. (2016)
			Williams et al. (2016)
			van Dam et al. (2017)
*Fusarium* sp. *radicis-cucumerinum*	fungus (Ascomicota)	increased pathogenicity	Ma et al. (2010)
			Balesdent et al. (2013)
			van Dam et al. (2017)
*Alternaria alternata*	fungus (Ascomicota)	increased pathogenicity caused by production of host-specific toxins	Hatta et al. (2002)
			Akagi et al. (2009)
			Ma et al. (2010)
			Balesdent et al. (2013)
			van Dam et al. (2017)
*Cochliobolus heterostrophus*	fungus (Ascomicota)	increased pathogenicity	Ma et al. (2010)
			Balesdent et al. (2013)
			van Dam et al. (2017)
*Leptoshaeria maculans*	fungus (Ascomicota)	increased pathogenicity	Ma et al. (2010) Balesdent et al. (2013) van Dam et al. (2017)
*Avena sativa*	plant (Poaceae)	resistance to rust	Dherawattana and Sadanaga (1973)
*Lolium peremne*	plant (Poaceae)	higher survival rate	Williams (1970)
			Rees and Hutchinson (1974)
*Allium schoenoprasum*	plant (Amaryllidaceae)	boost of the germination rate	Holmes and Bougourd (1989) Plowman and Bougourd (1994)
*Secale cereale*	plant (Poaceae)	heat tolerance	Pereira et al. (2017)
*Tischeria ekebladella*	moth (Lepidoptera)	sex chromosome	Dalíková et al. (2017)
*Plutella xylostella*	moth (Lepidoptera)	sex chromosome	Dalíková et al. (2017)
*Cameraria ohridella*	moth (Lepidoptera)	sex chromosome	Dalíková et al. (2017)
*Dryas iulia*	butterfly (Lepidoptera)	sex chromosome	Lewis et al. (2021)
*Cacopsylla peregrina*	plant lice (Psylloidea, Homoptera)	sex chromosome	Nokkala et al. (2003)
*Astyanax mexicanus*	cavefish (Actinopterygii)	sex chromosome	Imarazene et al. (2021)
numerous species	passerine birds (Passeriformes)	germline-restricted chromosome	Torgasheva et al. (2019)
			Kinsella et al. (2019)

A well-known case where a B chromosome is beneficial for its carrier can be seen in the chive plant *Allium schoenoprasum.* It has been observed that individuals with B chromosomes have better survival rates in their natural habitats than those without them. These B chromosomes have a positive effect on the development from seeds as they boost the germination rate in drought conditions (Holmes and Bougourd, 1989; Plowman and Bougourd, 1994) It is interesting that in *A. schoenoprasum* no mechanisms of drive have been found (Bougourd and Parker, 1979; Holmes and Bougourd, 1989), suggesting that the presence of B chromosomes in a population may be maintained by their positive effect on the carrier. A higher survival rate of plants with B chromosome compared to those without it was observed also in ryegrass, *Lolium peremne* (Rees and Hutchinson, 1974). In rye a comparison between individuals with and without B chromosomes suggests that rye B chromosomes may play a role in heat tolerance and may protect plants against damage caused by heat stress (Pereira et al., 2017).

In the fungus *Nectria haematococca,* resistance to antibiotics, which are naturally produced by pea plants, is associated with the presence of B chromosomes which show stable inheritance under certain conditions (Miao et al., 1991; Enkerli et al., 1997). These B chromosomes thus increase the pathogenicity of their host. Observations of an increase in pathogenicity under the influence of a B chromosome has also been demonstrated in other fungi species e.g., in *Magnaporthe oryzae*, *Fusarium oxysporum*, *F.* sp. *radicis-cucumerinum, Alternaria alternata, Cochliobolus heterostrophus* and *Leptoshaeria maculans* (Ma et al., 2010; Balesdent et al., 2013; van Dam et al., 2017). Interestingly, sequencing of the fungal B chromosomes revealed that these chromosomes display different genomic properties compared to the A chromosomes, including faster evolutionary rates, higher density of transposable elements and more gene duplications (Vanheule et al., 2016; Yang et al., 2020). It has been suggested that such a “two-speed” genome may bring an advantage to the pathogens by allowing more rapid adaptations to the host and new environments through more frequent mutations on the faster evolving B chromosomes (Croll and McDonald 2012). B chromosomes can also increase the resistance of their host to pathogens, such as, for example, in the oat plant, *Avena sativa,* where they increase the resistance of the plants to rust caused by the fungus *Puccinia coronata* f. sp. *avenae* (Dherawattana and Sadanaga, 1973).

It has been proposed that B chromosomes can become involved in sex determination and start to function as sex chromosomes (e.g., Camacho, 2005). Although, this crucial function can at the beginning represent a way how to enhance the B chromosome transmission as has been described in some cichlid fish (Clark and Kocher, 2019), it can later become essential for the host. In addition, the B chromosome can theoretically turn into a sex chromosome when it starts to pair with the X or Z in the system where Y or W is not present (i.e. X0 or Z0). Possible examples of such B to sex chromosome transition have been described in moth and butterfly species (Lepidoptera). Lukhtanov (2000) mentioned examples, which may represent different phases of such evolutionary transition, from the stage where a newly formed W chromosome (originally B chromosome) is still dispensable and individuals with or without it can be found in a population, to the stage where the W chromosome has the sex determining function and is fully fixed and essential. Although these examples support the possibility of the sex chromosome origin within B chromosomes, Lukhtanov (2000) provides alternative explanations of these observations. Also, in the family Tischeriidae and in the clade Ditrysia (Lepidoptera), the W possibly arose from a B chromosome which started to pair with the Z chromosome (Dalíková et al., 2017). However, a possibility that at least in Tischeriidae W chromosome evolved from the fusion of a Z chromosome and an autosome has not been ruled out (Dalíková et al., 2017; Hejníčková et al., 2019). Recent data from genome sequencing in another butterfly species *Dryas iulia* (tribe Heliconiini) support the origin of a W sex chromosome from a B chromosome and suggest that this event may have happened multiple times during the evolution of butterflies (Lewis et al., 2021). It has also been proposed that the ancestral Y chromosome in *Drosophila* may have originated from a B chromosome (Hackstein et al., 1996; Carvalho, 2002). None of the Y-linked genes in *Drosophila* have homologs on the X chromosome and all identified paralogs are autosomal. This implies that the origin and evolutionary history of the Y chromosome is different than simply being a degenerated counterpart of the X chromosome and Carvalho (2002) proposed that it could have its origin in a B chromosome that became a segregational partner of the X chromosome in an X0 system. However, it is also possible that the present Y in *Drosophila* could be an outcome of a fusion of an ancestral Y chromosome with an autosome (Bachtrog, 2013). In the plant lice *Cacopsylla peregrina*, the Y chromosome has most likely evolved from a B chromosome that was integrated into a segregation system with the X chromosome and later became fixed in the karyotype (Nokkala et al., 2003). In some cases, such as in the cichlid fish from the tribe Oreochromini, the B chromosome fused with the original sex chromosomes and become a stable part of the genome (Conte et al., 2021). In the cavefish *Astyanax mexicanus* the B chromosome contains a gene which determines male sex and therefore the B chromosome functions as a sex chromosome with a dominant male determining role (Imarazene et al., 2021).

In passerine birds (Passeriformes), an extra chromosome has been observed in the germline. This germline-restricted chromosome (GRC) is excluded by programmed DNA elimination from somatic cells during early embryogenesis (Wang and Davis, 2014). The GRC was first described in the zebra finch (*Taenopygia guttata*) (Pigozzi and Solari, 1998), and it has been revealed recently that it is likely present in all passerine birds (Passeriformes) (Kinsella et al., 2019; Torgasheva et al., 2019). The GRC is present in two copies in oocytes forming a bivalent that undergoes recombination. In contrast, in spermatocytes there is only a single copy of this chromosome which forms a heterochromatic element that is eliminated from the nucleus during the first meiotic division (Schoenmakers et al., 2010; del Priore and Pigozzi, 2014). Camacho et al. (2000) suggested that the GRC may be originally a supernumerary B chromosome which acquired an essential function for birds, possibly a germline determining role, preventing its loss. Genomic analysis of the GRC in zebra finch revealed that similarly as in B chromosomes, most GRC-linked genes are paralogs to genes on A chromosomes, which have been subsequently added to the GRC during passerine evolution (Kinsella et al., 2019). Like B chromosomes, GRC shows high variability in size ranging from the largest chromosome in the karyotype in some species to microchromosome in others (Torgasheva et al., 2019). Dedukh and Krasikova (2021) pointed out yet another similarity which can be found in the programed GRC elimination from somatic cells which strongly resembles mechanisms of tissue-specific B chromosome elimination as described in goatgrass *Aegilops speltoides* where the B chromosome is eliminated from roots (Ruban et al., 2020).

### Evolutionary Dynamics of B Chromosomes

Originally, two theoretical models were proposed to explain the occurrence of B chromosomes in populations and their maintenance in relatively stable frequencies. The first model assumed the spread of B chromosomes by some selfish drive mechanisms, opposed by negative effects of the B chromosomes on the carrier’s fitness if they are in high copy numbers (Jones 1995; Camacho et al., 2000). The second model (White, 1973) proposed that B chromosomes may be maintained in the population without drive mechanisms if they have a beneficial effect on their carriers in small numbers but start to be detrimental in high copy numbers. Empirical data reviewed in this paper supports both models providing evidence for the selfish spread of B chromosomes in populations through drive in many species (Hasegawa, 1934; Nur, 1962; Nur, 1963; Rutishauser and Rӧthlisberger, 1966; Nur, 1969; Kayano, 1971; Jones and Rees, 1982; Gregg at al., 1984; Murray, 1984; Viseras et al., 1990; Jones, 1991; Pardo et al., 1995; Houben, 2017; Jones, 2018; Clark and Kocher, 2019; Blavet et al., 2021) as well as identifying beneficial effects of B chromosomes for their hosts (Williams, 1970; Dherawattana and Sadanaga, 1973; Rees and Hutchinson, 1974; Holmes and Bougourd, 1989; Miao et al., 1991; Plowman and Bougourd, 1994; Enkerli et al., 1997; Hatta et al., 2002; Nokkala et al., 2003; Rodriguez-Carres et al., 2008; Akagi et al., 2009; Coleman et al., 2009; Ma et al., 2010; Balesdent et al., 2013; Thatcher et al., 2016; Williams et al., 2016; Dalíková et al., 2017; Pereira et al., 2017; van Dam et al., 2017; Armitage et al., 2018; Kinsella et al., 2019; Torgasheva et al., 2019; Imarazene et al., 2021; Lewis et al., 2021). From the example in rye where B chromosomes have beneficial function but are still driving (Pereira et al., 2017) we see that there could be even co-occurrence of drive and beneficial function which indicates the rather extensive complexity of B chromosome evolution.

As Camacho et al. (1997) suggested, parasitic B chromosomes initially spreading in a population by drive may be neutralized by the evolution of drive suppressors located on the A chromosomes if they harm the hosts. Since the elimination of already common B chromosomes from the population may be slow, the new drive genes may arise on the B before this elimination leading to a new cycle of B chromosome accumulation. The dynamics between B chromosomes and the rest of the genome may thus resemble a classical arms race between a parasite and its host. Certain studied species seem to show this type of dynamic where A chromosomes try to suppress the accumulation of B chromosomes and B chromosomes try to escape this pressure (Bosemark, 1954; Carlson, 1969; Nur and Brett, 1985, Shaw and Hewitt, 1985; Shaw et al., 1985; Nur and Brett, 1987, Nur and Brett, 1988; Romera et al., 1991; Cebriá et al., 1994; Jiménez et al., 1995; Herrera et al., 1996; Rosato et al., 1996; reviewed in Shaw and Hewitt, 1990; Camacho et al., 2000).

The examples collected in this review also suggest that sometimes B chromosomes can become stable and essential parts of the genome by gaining some vital function such as a role in sex determination or germline specific function. In fact, B chromosomes may be predisposed to become sex chromosomes or germline-restricted chromosomes by their selfish mechanisms of transmission including sex-specific meiotic drive and gonotaxis. In some cases, B chromosomes can also become accommodated into the genome by being translocated to autosomes or sex chromosomes as observed in several organisms (e.g., grasshoppers and maize) or could acquire regular behavior during meiosis, when two B chromosomes start to pair, both of which ensures their stable inheritance and maintenance in the population (e.g., Houben et al., 2000; Cabrero et al., 2003; Hsu et al., 2003).

## Conclusion

This review aims to portray B chromosomes as highly dynamic elements, with variable effects on their hosts, and rich evolutionary pathways. We are demonstrating that, although it was originally believed that B chromosomes behave mostly as genomic parasites with neutral or negative effects on the host being spread in the population by selfish drive mechanisms, a growing number of studies have shown that they can also have a positive effect on their hosts. Here we collected such examples including cases where B chromosomes increase the pathogenicity of their hosts or increase the survival rate in particular habitats. Moreover, we provide examples where B chromosomes likely became a stable and essential part of the genome by turning into new sex chromosomes or germline-restricted chromosomes. In this light, B chromosomes can be viewed as a reservoir of genetic material for the evolution of important genomic novelties with potentially significant evolutionary impacts.
